# Electrochemical Detection of SMN Protein by Immunosensors: The Role of Surface Modifications in Screen-Printed Carbon Electrodes

**DOI:** 10.3390/s26165171

**Published:** 2026-08-15

**Authors:** Mariana Rost Meireles, Giovana Dalpiaz, Muriel Schiling Krohn, Thuany Garcia Maraschin, Willyan Hasenkamp Carreira, André Anjos da Silva

**Affiliations:** 1 Institute of Technology for Food and Health (itt Nutrifor), Universidade Do Vale Do Rio Dos Sinos—Unisinos, São Leopoldo 93022-154, RS, Brazil; 2 Chemistry Post-Graduation Program, Universidade Federal Do Rio Grande Do Sul—UFRGS, Porto Alegre 91540-000, RS, Brazil; 3 Program in Materials Engineering and Technology, Pontifícia Universidade Católica Do Rio Grande Do Sul—PUCRS, Porto Alegre 90619-900, RS, Brazil; 4 Biosens Tecnologia para Saúde LTDA, São Leopoldo 93022-715, RS, Brazil; 5 Medical Science Post graduation Program, Universidade Do Vale Do Rio Dos Sinos—Unisinos, São Leopoldo 93022-154, RS, Brazil; 6 School of Medicine, Universidade Do Vale Do Taquari—Univates, Lajeado 95914-014, RS, Brazil

**Keywords:** genetic rare disorder, electrochemical immunosensor, screen-printed electrodes, antibody–antigen binding, innovative biosensing technologies, point-of-care diagnostics

## Abstract

**Highlights:**

**What are the main findings?**

- A gold electrodeposition procedure improved electrochemical performance of SPCEs and promoted enhanced electron transfer.
- The proposed SPCE-AuNS immunosensor differentiated specific (anti-SMN/SMN) from non-specific interactions through a pronounced electrochemical blocking effect evidenced by DPV measurements.

**What are the implications of the main findings?**

- The proposed platform demonstrates potential for future adaptation as a decentralized point-of-care strategy for Spinal Muscular Atrophy screening and monitoring.

**Abstract:**

Point-of-care (POC) technologies are promising tools to decentralize and accelerate the diagnosis of rare diseases. Among them, electrochemical immunosensors offer advantages such as high sensitivity, low cost, portability, low sample consumption, and suitability for use in resource-limited settings. However, the performance of these devices is dependent on electrode surface properties, which influence electron transfer, biomolecule immobilization, and analytical sensitivity. In this work, screen-printed carbon electrodes (SPCEs) were modified through two strategies: (i) gold electrodeposition and (ii) cold plasma treatment. The modified electrodes were functionalized with EDC/NHS, followed by the immobilization of anti-SMN antibodies and electrochemical characterization using cyclic voltammetry and differential pulse voltammetry. The impact of each modification approach on the electrochemical response and reproducibility of the sensor was evaluated. Gold electrodeposition resulted in higher and more reproducible electrochemical responses, demonstrating improved electron transfer properties and surface homogeneity. The primary objective of this study was to investigate how different surface modification strategies affect the electrochemical performance of SPCE-based immunosensors, employing the detection of Survival Motor Neuron (SMN) protein, a biomarker associated with Spinal Muscular Atrophy (SMA), as a proof-of-concept application. The resulting platform successfully differentiated specific and non-specific protein recognition events through distinct electrochemical patterns, demonstrating the suitability of gold-modified SPCEs for immunosensing applications. These findings provide insights into the influence of surface engineering strategies on sensor performance and support the future development of optimized electrochemical platforms for biomarker detection.

## 1. Introduction

Spinal Muscular Atrophy (SMA-5q) is a rare genetic and neurodegenerative disease characterized by the progressive loss of alpha motor neurons located in the spinal cord, resulting in muscle weakness and atrophy, respiratory failure, and, in the most severe cases, premature death [1]. SMA type I (infantile form) accounts for about 60% of cases and manifests in the first months of life, being the main monogenic cause of infant death [2]. SMA type I affects approximately 1 to 2 in every 100,000 live births, being included in the list of diseases considered rare (RD) [3], which are those that affect up to 5 in every 100,000 people (European Union metric) [4].

The disease is caused by mutations in the SMN1 gene (Survival Motor Neuron 1), located on chromosome 5q13, responsible for the production of the SMN protein, essential for the assembly of ribonucleoprotein complexes and for the maintenance of motor neurons [5]. A homologous gene, SMN2, partially compensates for SMN production; however, exon 7 skipping results in the generation of a predominantly unstable and truncated protein form [6,7]. Therefore, SMA patients show lower levels of SMN on blood samples, turning this protein into a potential molecular biomarker for diagnosis and monitoring [8,9].

Despite recent therapeutic advances, such as nusinersen, risdiplam, and onasemnogene abeparvovec gene therapy, which raise SMN levels and improve prognosis [10], effectiveness depends on early initiation of treatment, ideally before the onset of symptoms [11]. Current approaches involve the detection of SMN1 deletion or intragenic mutations through biomolecular tests such as Multiplex Ligation Probe Amplification (MLPA) and gene sequencing, which require complex laboratory structures [12].

Point-of-care (POC) technologies present themselves as a viable alternative to decentralize and expedite the diagnosis of rare diseases. Specifically, electrochemical immunosensors are widely used in studies to develop immunological assays with high sensitivity, low cost, the ability to use small sample volumes, and easy application in different contexts. POC devices are portable and can be applied in regions with difficult access [13], highlighting the possibility of screening patients for SMA in remote parts of the world. Although POC tests can already present advantages such as portability, low sample consumption, and rapid analysis compared to traditional immunological assays, this performance can be further improved through surface modification. Strategies such as cold plasma treatment and gold electrodeposition enhance sensitivity by increasing surface functional groups, improving wettability, potentially improving electrochemical performance and biomolecule immobilization, and expanding the electroactive area available for biomolecule immobilization on screen-printed carbon electrodes (SPCEs) [14,15]. Conventional immunological assays such as ELISA generally provide excellent analytical sensitivity and lower limits of detection, but they require specialized laboratory infrastructure, trained human resources, and centralized analysis [16]. Conversely, lateral flow assays (LFAs) offer portability, simplicity, and agility, although they are typically limited to qualitative or semi-quantitative measurements. On the other hand, electrochemical point-of-care platforms show portability and the potential for quantitative biomarker detection with agility and low sample consumption [17,18].

This study aims to contribute to advancing the use of a point-of-care electrochemical device based on the detection of the SMN protein (a biomarker that is absent or reduced in patients with SMA), using a specific antibody. This will support studies not only on decentralized neonatal screening for SMA, but also on therapeutic monitoring, since SMN concentration can reflect the response to treatment.

Therefore, the primary objective of this study was to compare two surface modification strategies for screen-printed carbon electrodes, namely oxygen cold plasma treatment and gold electrodeposition, and to evaluate their influence on electrochemical performance, reproducibility, and biomolecule recognition. The detection of the Survival Motor Neuron (SMN) protein was employed as a proof-of-concept biological application to assess the suitability of the modified interfaces for immunosensing purposes. By establishing the relationship between surface engineering and electrochemical behavior, this work aims to contribute to the development of optimized SPCE-based biosensing platforms for future biomarker detection applications. Considering the clinical relevance of SMN and the limited worldwide implementation of SMA screening programs, with less than 2% of newborns currently screened according to Dangouloff et al. [19], the development of accessible and decentralized detection platforms remains an important long-term objective.

## 2. Materials and Methods

**Figure 1:**
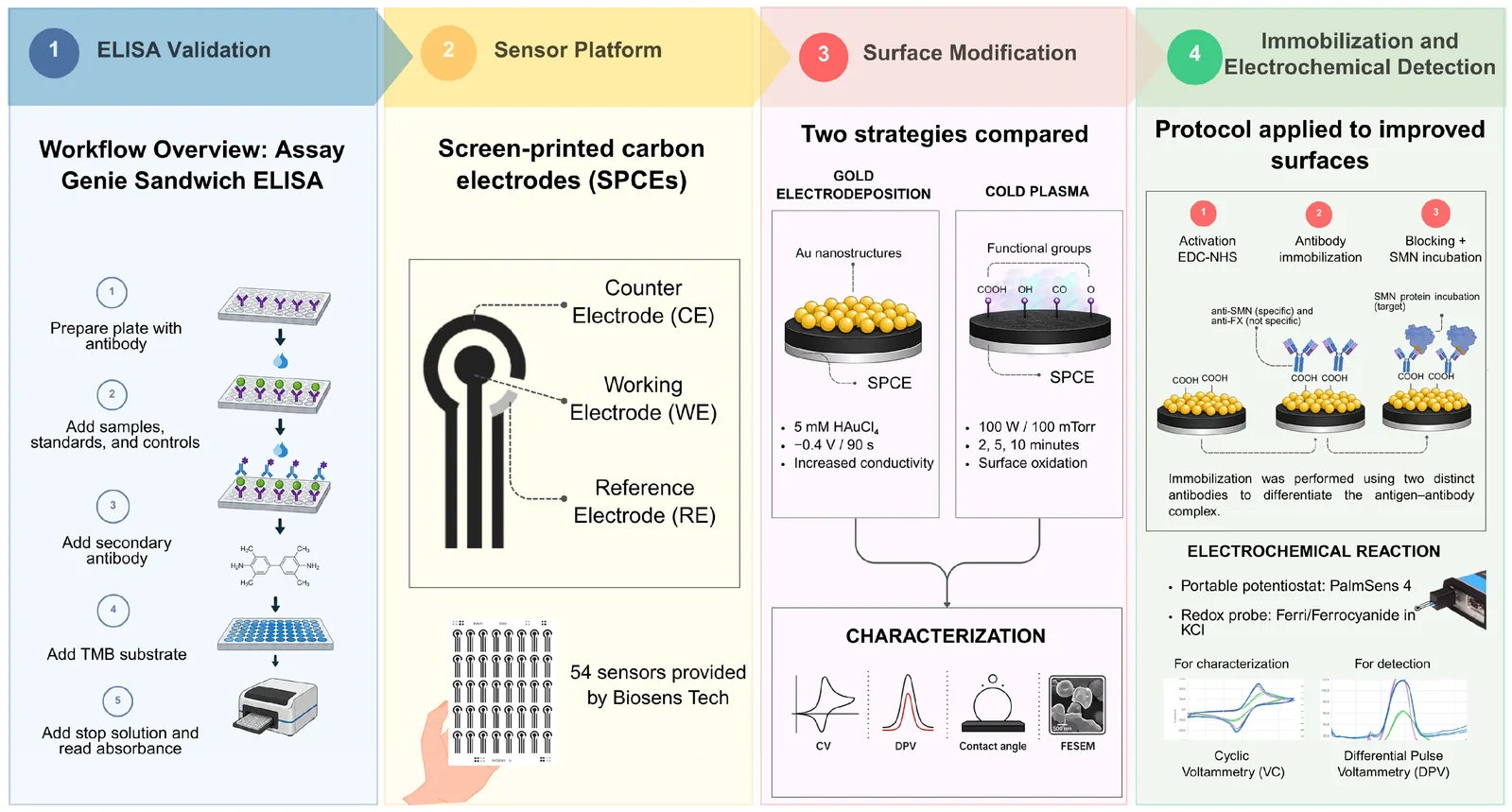
Workflow of the development and evaluation of the electrochemical immunosensor for SMN protein detection. The study approach was organized into four main stages: (1) biomarker detection validation using ELISA, (2) sensor platform selection, (3) surface modification strategies, (4) immobilization process and electrochemical characterization and immunosensor detection using cyclic voltammetry (CV) and differential pulse voltammetry (DPV).

The methodology for sensor development and biomolecule detection is outlined in Figure 1 for clarity. The procedures include: the selection of sensors and materials (Section 2.1), biomarker detection validation using Enzyme-Linked Immunosorbent Assay (ELISA) (Section 2.2), sensor surface modification through cold plasma treatment and gold electrodeposition (Section 2.3), and antibody immobilization followed by electrochemical characterization and target protein detection (Section 2.4).

### 2.1. Sensors and Materials

This study involved the validation of SMN protein as a biomarker using a Human SMN1 Sandwich ELISA Kit (AssayGenie HUFI02863, Dublin, Ireland), containing a 96-well microplate, lyophilized standard, sample dilution buffer, biotin-labeled antibody, HRP (horse-radish peroxidase)–streptavidin conjugate, dilution buffer, tetramethylbenzidine (TMB) substrate, stop solution, and wash buffer.

For electrochemical sensor development, a total of 54 screen-printed carbon electrodes (SPCEs) made by graphene–carbon paste were used. All sensors were provided through a research partnership with BIOSENS Tecnologia em Biossensores Ltda., São Leopoldo, RS, Brazil. Each SPCE consisted of a three-electrode configuration, comprising a counter electrode (CE), a working electrode (WE) where the immobilization procedures were performed, and a carbon-based pseudo-reference electrode (RE).

The bioreceptor immobilizing procedure was performed by employing 1-ethyl-3-(3-dimethylaminopropyl)carbodiimide (EDC) and N-hydroxysuccinimide (NHS) at a 1:1 ratio. This activation step was carried out in the MES buffer (2-(N-morpholino)ethanesulfonic acid, 25 mM, pH 5.0). For blocking and washing steps, 0.125% TTBS-BSA (Tris-buffered saline containing Tween-20 and bovine serum albumin) and 0.05% TBS-T (Tris-buffered saline with Tween-20) were used to minimize non-specific binding and ensure specific recognition of target biomolecules. For the capture step, negative sensors employed anti-FX/FXa antibodies (F8396, Sigma-Aldrich, Sigma-Aldrich, St. Louis, MO, USA) as non-specific bioreceptors at a 2 μg/mL concentration, while positive ones used anti-SMN antibodies (ADI-NBA-202; Enzo Life Sciences, Farmingdale, NY, USA) as bioreceptors, also at 2 μg/mL. Both were immobilized onto the WE activated surface. The SMN protein (ADI-NBP-201, Enzo Life Sciences, Farmingdale, NY, USA) was used as the target analyte for detection assays at a 0.36 μg/mL concentration.

Electrochemical measurements were performed using a portable potentiostat (PalmSens 4, PalmSens BV, Houten, The Netherlands). A ferricyanide solution prepared in Potassium Chloride (KCl) was used as the redox mediator to enable electrochemical characterization of the modified electrodes. Data acquisition and analysis were performed using PSTrace 5.11 software.

### 2.2. Biomarker Detection Validation Using ELISA

ELISA was applied to evaluate the binding capacity between the anti-SMN antibody and its antigen SMN protein, considering immunological response and antigen–antibody complex formation. The lyophilized standard was reconstituted with sample dilution buffer to a stock concentration of 2 ng/mL. A serial dilution of standard was performed to obtain final concentrations of 1 ng/mL, 0.5 ng/mL, 0.25 ng/mL, 0.125 ng/mL, 0.0625 ng/mL, 0.0316 ng/mL, and 0.0106 ng/mL. Subsequently, 100 μL of each dilution was added to individual wells and incubated at 37 °C for 90 min. The plate was washed twice with 350 μL of wash buffer, followed by biotin-labeled antibody addition (100 μL) and incubated at 37 °C for one hour. The washing procedure was repeated three times, and 100 μL of HRP–streptavidin solution was added to each well, followed by additional washing processes in the same conditions as before. Then, 90 μL of TMB substrate was added, and the plate was incubated in the dark for 15 min (37 °C). After that, 50 μL of stop solution was added to each well. The absorbance pattern was measured at 450 nm using a microplate reader and obtained data was analyzed using the Rplatform.

### 2.3. Sensor Modification Procedures

Two different modification strategies were applied to the SPCEs to enhance their surface properties and promote efficient immobilization of the bioreceptor: cold plasma treatment and gold electrodeposition. Both approaches were compared to determine which modification most effectively facilitated antibody attachment and, consequently, the detection of the target biomolecule.

#### 2.3.1. Cold Plasma Treatment

Cold plasma treatment was conducted using a Plasma ETCH system to oxidize the graphene surface of the electrodes, thereby generating functional groups suitable for antibody immobilization. Treatments were performed at 150 W and 100 mTorr for varying durations (2, 5, and 10 min) to determine the optimal exposure time for effective surface modification, while avoiding potential structural degradation associated with prolonged plasma exposure [20].

#### 2.3.2. Gold Electrodeposition

SPE surfaces were modified via gold electrodeposition to improve their electrochemical performance. This procedure involved applying 100 μL of a 5 mM gold(III) chloride hydrate (HAuCl_4_) solution onto the working electrode surface, followed by the application of a constant potential of −0.4 V for 90 s [21]. After electrodeposition, the electrodes were thoroughly rinsed with deionized water and dried through adsorption to remove any residual reagents, preparing them for subsequent functionalization.

#### 2.3.3. Evaluation

The performance of both modification methods was assessed using electrochemical techniques, including cyclic voltammetry (CV) and differential pulse voltammetry (DPV). In addition, contact angle measurements were performed to evaluate changes in surface hydrophobicity/hydrophilicity resulting from the modifications. In this procedure, the angle formed between a water droplet and the electrode surface was measured to infer the wettability properties, which are critical for efficient bioreceptor immobilization. Images of the contact angles (.jpeg or .png) were analyzed using ImageJ software to quantify the surface characteristics and guide the selection of the most suitable modification method. Measurements were performed in triplicate for each condition and results are presented as mean ± standard deviation. Statistical comparisons among groups were performed using one-way analysis of variance (ANOVA) followed by Tukey’s post hoc test, with *p* < 0.05 considered statistically significant.

Based on electrochemical and physicochemical results, gold-electrodeposited sensors were also evaluated using field emission scanning electron microscopy (FESEM), enabling the assessment of the morphology of the nanostructures formed during the procedure. Images were acquired using an Inspect F50 (FEI) microscope (FEI Company, Hillsboro, OR, USA) operated at an accelerating voltage of 20 kV, allowing for high-resolution visualization.

To facilitate a direct comparison between the applied surface modification strategies, their key characteristics, operational parameters, and expected impacts on sensor performance are summarized in Table 1.

**Table 1:** Comparison between the gold electrodeposition and cold plasma treatment strategies employed for surface modification.

Parameter	Gold Electrodeposition	Cold Plasma Treatment
Principle	Electrochemical reduction of Au^3+^ ions forming metallic nanostructured layer [22]	Surface oxidation via ionized gas generating functional groups [20]
Objective	Increase conductivity and electroactive surface area [23]	Introduce oxygen-containing functional groups for biomolecule anchoring [20]
Reagent/System	HAuCl_4_ (5 mM)	Plasma ETCH system
Key conditions	−0.4 V for 90 s; 100 µL solution	100 W, 100 mTorr; 2, 5, and 10 min
Surface effect	Formation of gold nanostructures (AuNSs)	Increased surface oxidation and functionalization (e.g., –OH, –COOH)
Impact on immobilization	Enhances electron transfer and increase electroactive interface	Improve surface wettability and chemical anchoring potential
Characterization methods	VC, DPV, FESEM and contact angle	VC, DPV, contact angle

### 2.4. Immobilization Procedures and Detection

In this section, the procedures for antibody immobilization aiming to detect SMN protein are described. The experimental strategy includes characterization of the modified electrode surfaces, evaluation of immobilization efficiency, and the use of appropriate controls to assess specificity.

#### 2.4.1. Antibodies Immobilization and Target Protein Detection

The immobilization protocol was carried out in three sequential steps and was applied exclusively to the electrode surface modified by the strategy that demonstrated the best performance in the physicochemical and electrochemical characterizations. (i) Initially, 20 μL of an EDC/NHS solution (1:1) was applied to the WE after the surface modification step to activate the electrode surface for bioreceptor attachment. This reagent enables the immobilization of biomolecules, particularly proteins, through the interaction between activated carboxyl groups and amino groups presented in the antibody structure. The modified SPCEs were incubated overnight under controlled humidity conditions, leading to completely dry SPCEs through the use of silica gel, thereby mitigating the risk of NHS hydrolysis. (ii) Subsequently, SPCEs were washed using 30 μL of MES buffer, followed by rinsing with deionized water to remove unreacted reagents. Then, 20 μL of the capture antibody was immobilized onto the electrode surface (anti-SMN for the specific recognition condition (considered as positive) and anti-FX for the non-specific control (negative)). An overnight incubation under controlled humidity was then carried out. (iii) The WE surface was then blocked with 20 μL of 0.125% TTBS-BSA for 2 h at 25 °C to minimize non-specific interactions. After the blocking step, 20 μL of the SMN protein solution was applied to both experimental conditions and incubated for 3 h at 25 °C. Finally, the electrodes were washed with 30 μL of 0.05% TBS-T, followed by rinsing with deionized water, to remove unbound molecules.

#### 2.4.2. Electrochemical Measurements and Data Analysis

The SPCEs’ electrochemical behavior was characterized using two techniques: cyclic voltammetry (CV) and differential pulse voltammetry (DPV). Measurements were conducted at distinct stages of the study: initially to evaluate and select the most effective surface modification strategy, and subsequently to evaluate antigen–antibody complex formation and its effect in electrochemical measurements. For this purpose, different sensor conditions were analyzed, including bare electrodes, electrodes modified with either anti-SMN or anti-FX antibodies only, and electrodes after exposure to the target antigen (SMN protein), enabling comparison according to specific antibody recognition systems.

CV was employed to investigate the redox activity of the system and to assess changes in the electrochemical profile at each modification step, which are indicative of surface alterations and biomolecular interactions. DPV was selected due to its high sensitivity when detecting changes in faradaic current associated with surface modification, biomolecule immobilization, and antigen–antibody interactions, enabling accurate evaluation of the analytical performance of the sensor. Furthermore, the technique allows the extraction of multiple peak-related parameters, providing a comprehensive assessment of electrochemical performance and interfacial changes. CV measurements were performed over 10 consecutive cycles, using an initial potential of 0.0 V, a potential step of 0.01 V, and a scan rate of 0.05 V/s. The fifth cycle was selected for analysis and comparison, as the current–potential profile stabilized after the initial scans, in agreement with previous reports, minimizing transient effects and ensuring reproducibility across measurements [24].

DPV measurements were carried out using a potential window from −0.5 V to 0.5 V, with a potential step of 0.01 V, pulse amplitude of 0.05 V, pulse time of 0.1 s, and scan rate of 0.02 V/s. All measurements were performed using 20 μL of a ferri/ferrocyanide redox probe solution. Each condition was evaluated in triplicate using independent sensors to avoid surface degradation caused by repeated exposure to the redox mediator, minimize inter-sensor bias, and ensure the robustness of the sensing platform.

For data analysis, DPV peak parameters including peak current (μA), peak potential (V), peak width (V), peak area (μA·V), maximum slope (μA/V), minimum slope (μA/V) and sum slope (μA/V) were extracted. In addition to peak current, peak area (µA·V) was evaluated because it considers both peak height and width turning possible to comprehend if variations in electrochemical signal are related to peak intensity, peak broadening, or both, providing complementary information regarding the effects of surface modification on the electrochemical interface. Slope-related parameters were extracted as complementary descriptors of the voltammetric peak profile. Although related to peak height and width, they provide an additional quantitative assessment of peak geometry, supporting the interpretation of electrochemical changes induced by surface modification and biomolecular recognition. From CV measurements, anodic (I_pa_) and cathodic (I_pc_) peak currents, along with their corresponding peak potentials (E_pa_ and E_pc_), were obtained. The peak-to-peak separation (ΔE) and the peak current ratio (I_pa_/I_pc_) were subsequently calculated. These parameters are commonly used to evaluate the reversibility of the redox process and the electron transfer kinetics of an electrochemical system.

## 3. Results and Discussion

### 3.1. Validation of SMN Protein Detection by an ELISA

**Figure 2:**
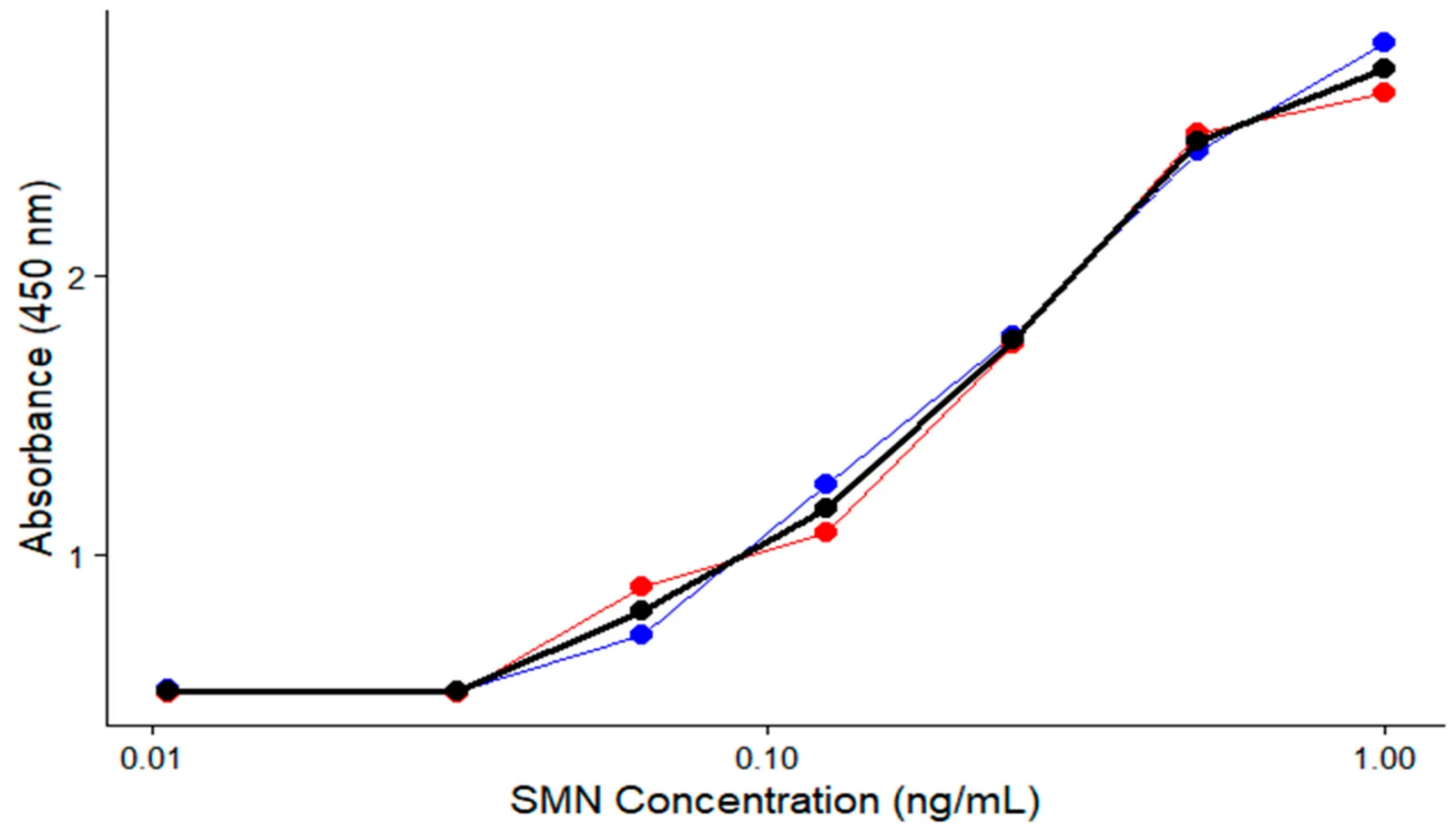
Validation of SMN protein detection through ELISA: absorbance values obtained for serial dilutions of the recombinant SMN standard, ranging from 0.0106 to 1 ng/mL, along with the negative control. The blue and red lines represent independent duplicate measurements, while the black line indicates mean absorbance for each concentration. Increasing SMN concentrations resulted in progressively higher absorbance values, confirming concentration-dependent antigen–antibody recognition.

Before electrochemical analysis, the complex formation and anti-SMN antibody recognition system was validated through a conventional Enzyme-Linked Immunosorbent Assay (ELISA). The SMN standard provided in the ELISA kit was reconstituted in 1 mL of sample dilution buffer, resulting in a stock concentration of 2 ng/mL. Serial dilutions were then prepared to evaluate the assay response across a range from 0.0106 to 1 ng/mL. Each dilution was evaluated in duplicate to confirm the recognition behavior among different concentrations. Figure 2 demonstrates the absorbance variation among the SMN dilutions, evidencing that increasing concentrations resulted in progressively higher absorbance values, which demonstrates effective antigen–antibody recognition and confirms the suitability of the selected immunological system.

A progressive reduction in signal intensity was observed with decreasing SMN concentrations. Absorbance values tended to stabilize at lower concentrations (≤0.0313 ng/mL), possibly reflecting a reduced assay response near the lower concentration range. Nevertheless, detectable signals were maintained even at low protein concentrations, supporting the feasibility of adapting the antibody–SMN recognition system to an electrochemical sensing platform.

### 3.2. Surface Sensor Modifications Contribution for Increase Electrochemical Performance

The effects of cold plasma and gold electrodeposition treatments were evaluated through contact angle measurements, as well as CV and DPV analyses. Initially, different cold plasma exposure times were investigated, with the best electrochemical performance achieved after 2 min of treatment. This optimized condition was subsequently compared with gold electrodeposition, which demonstrated superior reproducibility and higher current responses, indicating improved electron transfer properties. Therefore, although both surface modification strategies increased the hydrophilicity of the sensor surface and improved analytical sensitivity, gold electrodeposition proved to be the most advantageous pretreatment approach.

#### 3.2.1. Determining Cold Plasma Exposure Time Through Electrochemical Measurements

Aiming to understand the effect of O_2_ cold plasma exposure time on sensor surface properties and electrochemical behavior, SPCEs were submitted to three different treatment time conditions (2, 5, and 10 min). The electrochemical response to each condition was characterized using CV and DPV, with the intent to recognize the most stable, sensitive, and reproducible surface modification condition.

**Figure 3:**
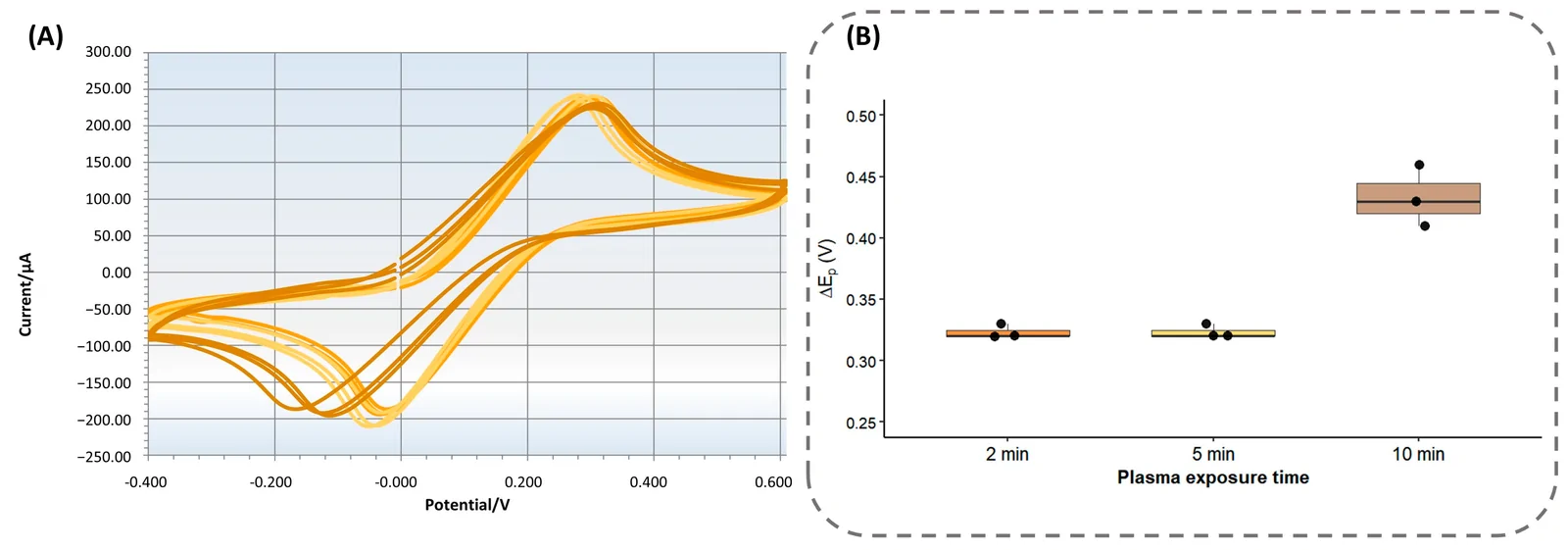
Plasma exposure time evaluation through the cyclic voltammetry (CV) electrochemical technique. (**A**) Cyclic voltammograms obtained for the three plasma exposure conditions (2, 5, and 10 min, represented in orange, yellow and soft brown, respectively). (**B**) The extracted electrochemical parameters for ΔE (V).

Considering the CV results (Figure 3A,B), plasma incubation time directly affects the electrochemical behavior of the sensing interface. The 2 and 5 min conditions exhibited similar electrochemical profiles, with comparable and approximate peak currents magnitudes (±240 µA) and ΔE values (~0.32 V), indicating preserved electron transfer kinetics. The same did not occur for the 10 min incubation time, which resulted in a pronounced increase in ΔE (~0.43 V) and reduction in anodic and cathodic current responses (about 240 µA). These results suggest that a 10 min treatment could consist of excessive plasma exposure that promotes overoxidation of the electrode interface, impairing electron transfer properties and reducing the electrochemical activity of the system. Supplementary Figure S1 shows the plots for cathodic and anodic peak potentials (E_pc_ and E_pa_), and also cathodic and anodic peak currents (I_pc_ and I_pa_).

**Figure 4:**
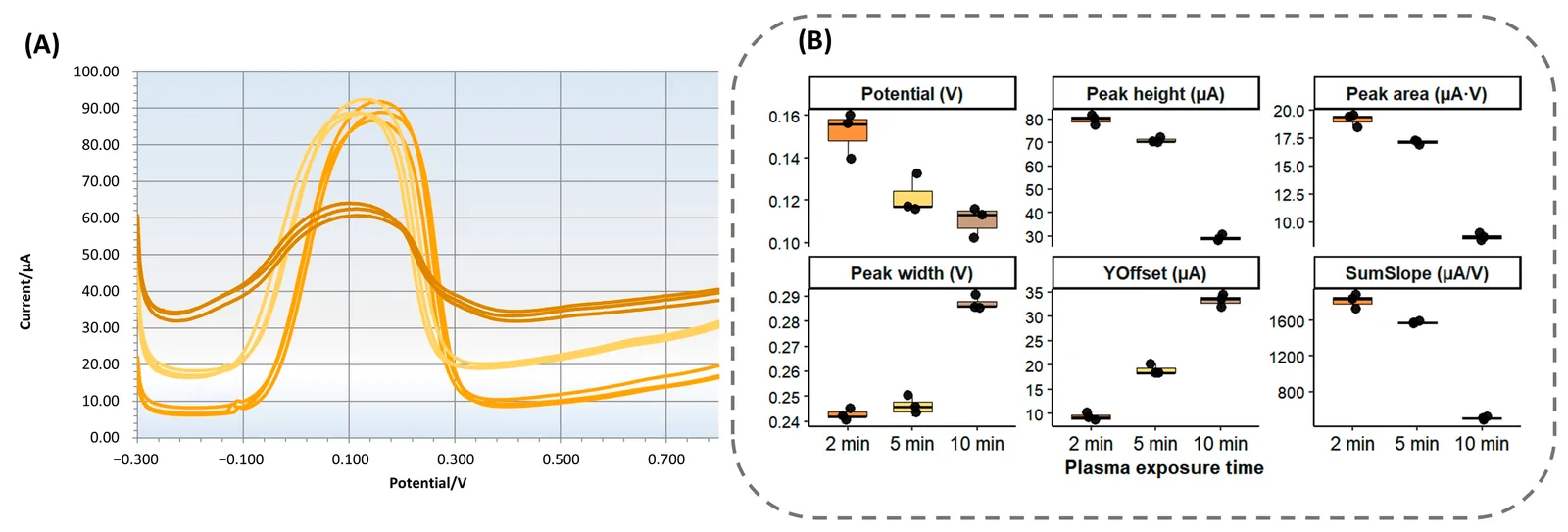
Plasma exposure time evaluation through the DPV electrochemical technique. (**A**) The voltammograms for the three plasma exposure conditions (2, 5, and 10 min, represented in orange, yellow and soft brown, respectively). (**B**) Extracted electrochemical parameters including potential (V), peak height (µA), area (µA·V), width (V), Yoffset (µA) and Sum Slope (µA/V). The higher slope-related values observed for the 2 min condition indicate improved electron transfer kinetics and enhanced electrochemical activity compared to longer plasma exposure times.

DPV results are presented in Figure 4, which confirm the influence of plasma incubation time on the sensors’ electrochemical behavior. The 2 min condition exhibited the highest peak current values (~80 µA), peak area (~19 µA·V), and SumSlope (~1819 µA/V), indicating enhanced electron transfer efficiency and superior electrochemical responsiveness [25,26]. Although the 5 min condition still maintained relatively elevated electrochemical responses, moderate reductions in peak current and slope-related parameters suggested the onset of interfacial blocking effects. The 10 min plasma incubation caused a substantial suppression of the electrochemical signal, with peak current values decreasing to approximately 29 µA and SumSlope values reduced by nearly 70% compared to the 2 min condition. Additionally, the increase in YOffset and peak width observed for the 10 min condition suggests enhanced capacitive contribution, surface heterogeneity, and progressive passivation of the electroactive interface [27]. The progressive reduction in peak area suggests that the electron transfer capacity decreases with longer treatment times. The combined decrease in both peak height and area indicates that increasing plasma exposure time leads to a loss of the sensor’s electroactive surface area. The reduction in SumSlope indicates a less pronounced peak profile, which together support the hypothesis that excessive plasma treatment compromises the electroactive surface and impairs charge transfer. Plots considering minimum and maximum slope are depicted in Supplementary Figure S2.

Considering both CV and DPV analyses, the 2 min plasma exposure condition was selected for subsequent experiments, as it provided the best balance between interfacial stability and analytical responsiveness. This condition was subsequently compared to the gold electrodeposition procedure and bare SPCEs to determine the most suitable surface modification strategy.

#### 3.2.2. Comparison Between Effects Generated by Plasma Exposure and Gold Electrodeposition Considering Electrochemical Behavior

The surface modification procedures were evaluated through CV and DPV measurements that revealed distinct aspects of the SPCEs’ electrochemical performance. A dataset with all values for analyzed parameters of CV and DPV from those and other conditions is provided in Supplementary Materials Tables S1 and S2.

**Figure 5:**
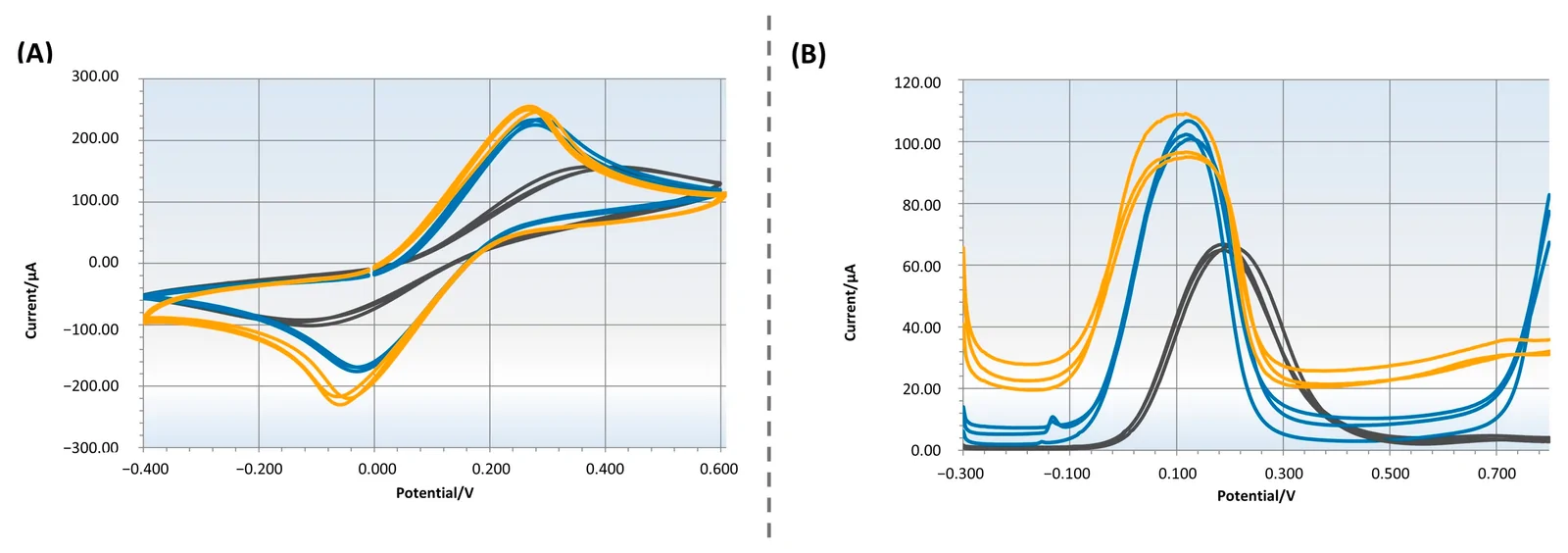
Comparison among electrochemical behavior of bare, plasma-treated, and gold-electrodeposited SPCEs. (**A**) Cyclic voltammetry (CV) responses and (**B**) differential pulse voltammetry (DPV) responses obtained for the three evaluated surface conditions. Black lines represent bare SPCEs, while blue lines represent gold-electrodeposited ones (SPCE-AuNS), and yellow lines represent plasma-treated (2 min exposure) ones. Both modification strategies promoted increased electrochemical activity compared with non-modified electrodes. Plasma treatment resulted in pronounced electrochemical activation, whereas gold electrodeposition exhibited improved signal reproducibility and more favorable electron transfer behavior.

CV measurements demonstrated that both plasma treatment and gold electrodeposition promoted an increase in anodic and cathodic current responses compared with bare (non-modified) electrodes, indicating enhanced electrochemical activity after surface modification (Figure 5A). Plasma-exposed electrodes exhibited slightly higher oxidation and reduction current magnitudes (about 244 µA and −246 µA, respectively) compared with gold-electrodeposited sensors (approximately 237 µA and −207 µA), suggesting enhanced electrochemical activation of the interface potentially associated with plasma-induced surface oxidation and increased surface reactivity [28,29]. However, despite the slightly higher current responses observed for plasma-treated electrodes, gold electrodeposition exhibited lower ΔE values (~0.30 V) compared with plasma-treated sensors (~0.33 V), indicating more favorable electron transfer kinetics and improved reversibility of the electrochemical system. These findings suggest that plasma treatment promoted stronger surface activation, whereas gold electrodeposition generated a more electrochemically efficient and stable interface.

**Figure 6:**
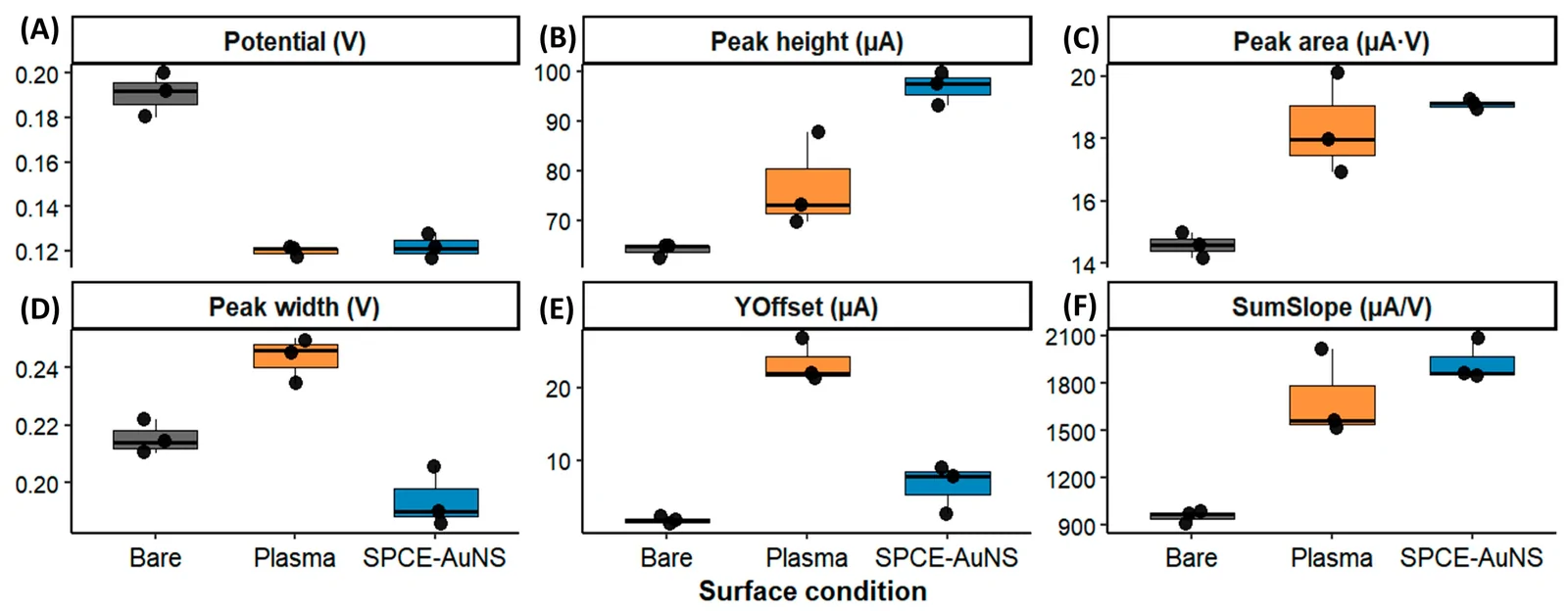
Effect of surface modification on the electrochemical response of SPCEs evaluated by differential pulse voltammetry (DPV) parameters: (**A**) peak potential, (**B**) peak height, (**C**) peak area, (**D**) peak width, (**E**) baseline current (YOffset), and (**F**) SumSlope. The parameters were obtained for bare (black), plasma-treated (yellow), and gold-electrodeposited (SPCE-AuNS—blue) SPCEs. Data are presented as individual measurements (*n* = 3) with mean values indicated. Gold electrodeposition resulted in higher and more consistent electrochemical responses, while plasma treatment showed increased variability and higher baseline currents.

Considering the DPV results, bare SPCEs exhibited lower peak current values, in the range of approximately 62–65 µA, while plasma-treated and electrodeposited SPCEs exhibited current ranges of ~70–88 µA and 93–100 µA, respectively, indicating an increase in current response and electrochemical activity. Considering cold plasma treatment, this effect could be due to surface activation with oxygen functional groups, while in gold electrodeposition, which exhibited higher and more consistent currents among the replications (Figure 5B and Figure 6A), the improvement is associated with an increase in electroactive surface due to gold nanostructure (AuNS) formation, improving electron transfer kinetics. Plasma treatment resulted in pronounced electrochemical activation, whereas gold electrodeposition exhibited improved signal reproducibility and more favorable electron transfer behavior [30]. This trend was also supported by the peak area (Figure 6C) analysis which reflects the overall charge transfer, with bare, plasma-treated and electrodeposited SPCEs exhibiting ranges of 14–15 µA/V, 17–20 µA/V and ~19 µA/V respectively. Although plasma-treated electrodes exhibit comparable or slightly higher peak area values, they are more dispersed compared to gold-electrodeposited ones, suggesting lower reproducibility.

Peak width analysis (Figure 6D) revealed notable differences in surface homogeneity and electrochemical behavior. Plasma-treated electrodes exhibited broader peaks (~0.23–0.25 V), suggesting increased heterogeneity and a wider distribution of electroactive sites. In contrast, gold-electrodeposited electrodes (SPCE-AuNSs) displayed narrower peaks (~0.19–0.21 V), indicative of a more homogeneous surface and electron transfer processes. SPCE-AuNS exhibited consistent electrochemical responses, suggesting improved electron transfer properties and increased electroactive surface area. These characteristics are particularly desirable for electrochemical biosensing applications. Also, considering YOffset values (Figure 6E), plasma-treated SPCEs exhibited a substantial increase in baseline current (21–27 µA) compared to bare (~1–2 µA) and electrodeposited (3–9 µA) ones, indicating a higher contribution of non-faradaic processes. This effect may reduce the signal-to-background ratio, which may compromise the effective signal discrimination despite the observed increase in peak current.

#### 3.2.3. Physical Effects of Plasma Exposure and Gold Electrodeposition Procedures

Contact angle measurements revealed a progressive and statistically significant decrease with increasing plasma exposure time, indicating a gradual enhancement in surface wettability promoted by oxygen plasma treatment (one-way ANOVA followed by Tukey’s post hoc test, *p* < 0.05), as shown in Supplementary Figure S3. Mean contact angle values decreased from approximately 91.9° for bare SPCEs to 32.6°, 21.3°, and 13.1° after 2, 5, and 10 min of plasma exposure, respectively. In contrast, gold electrodeposition resulted in a more moderate reduction (61.8°), indicating that its surface modification mechanism differs from that promoted by plasma treatment. The progressive increase in wettability is consistent with plasma-induced surface oxidation and the introduction of oxygen-containing functional groups, which increase water affinity and may favor biomolecule immobilization and molecular spreading at the electrode interface. Although the 10 min plasma treatment exhibited the lowest contact angle, it also produced the poorest CV and DPV responses. These findings suggest that maximizing surface wettability alone is insufficient to optimize sensor performance. Instead, prolonged plasma exposure likely promotes excessive oxidation of the graphene–carbon surface, compromising electron-transfer efficiency despite the favorable wetting properties. Despite the superior hydrophilic behavior, electrochemical analyses demonstrated that gold-electrodeposited SPCEs exhibited improved electron transfer behavior, lower baseline current, higher current responses, and better reproducibility.

**Figure 7:**
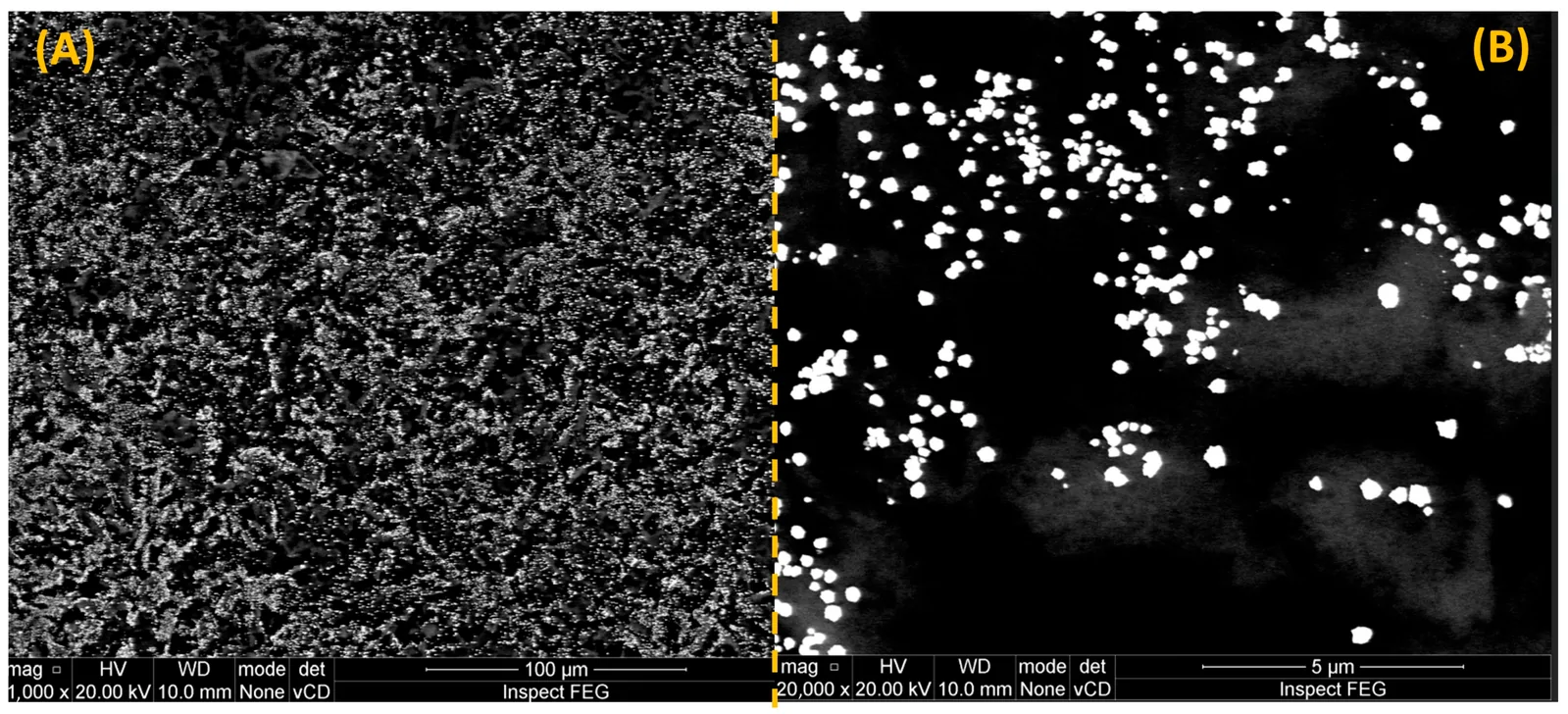
Field emission scanning electron microscopy (FESEM) images demonstrating the effect of gold electrodeposition on the sensor surface. (**A**) SPCE-AuNS working electrode (WE) at 1000× magnification and (**B**) at 20,000× magnification. The images confirm the formation and spatial distribution of gold nanostructures (AuNSs) on the sensor surface. Notably, AuNSs did not completely cover the working electrode surface, suggesting the formation of discrete conductive nanostructures rather than a continuous metallic layer.

The gold electrodeposition modification was also evaluated and confirmed by FESEM in order to investigate the distribution and morphology of gold nanostructure (AuNS) formation at the sensor surface. Figure 7 represents the WE of a SPCE-AuNS sample; it is important to notice that AuNSs did not completely cover the WE surface, suggesting the formation of conductive AuNSs rather than a continuous metallic layer. The presence of these nanostructures contributed to the enhanced electrochemical behavior observed in CV and DPV measurements, potentially by increasing the electroactive surface area and facilitating electron transfer at the electrode interface and maintaining the graphene–carbon interface’s intrinsic characteristics for immobilizing procedures. These findings support the selection of SPCE-AuNSs for subsequent immunosensor development.

It is important to emphasize that AuNSs do not covering the entire graphene matrix, as demonstrated in Figure 7B, keeping the original electrode surface available for functionalizing agents. Considering that EDC/NHS-mediated coupling is not expected to occur directly on metallic gold surfaces, the activation likely occurs on functional groups available in the remaining carbonaceous interface. Additionally, while covalent immobilization is expected to contribute to antibody attachment, the participation of non-covalent adsorption mechanisms on AuNS-modified surfaces cannot be completely excluded. Therefore, additional surface-sensitive characterization techniques would be valuable in future studies to further elucidate the relative contribution of covalent immobilization and physical adsorption on AuNS-modified electrodes.

### 3.3. Specific Detection of SMN Protein and Perspectives in SMA Diagnosis

**Figure 8:**
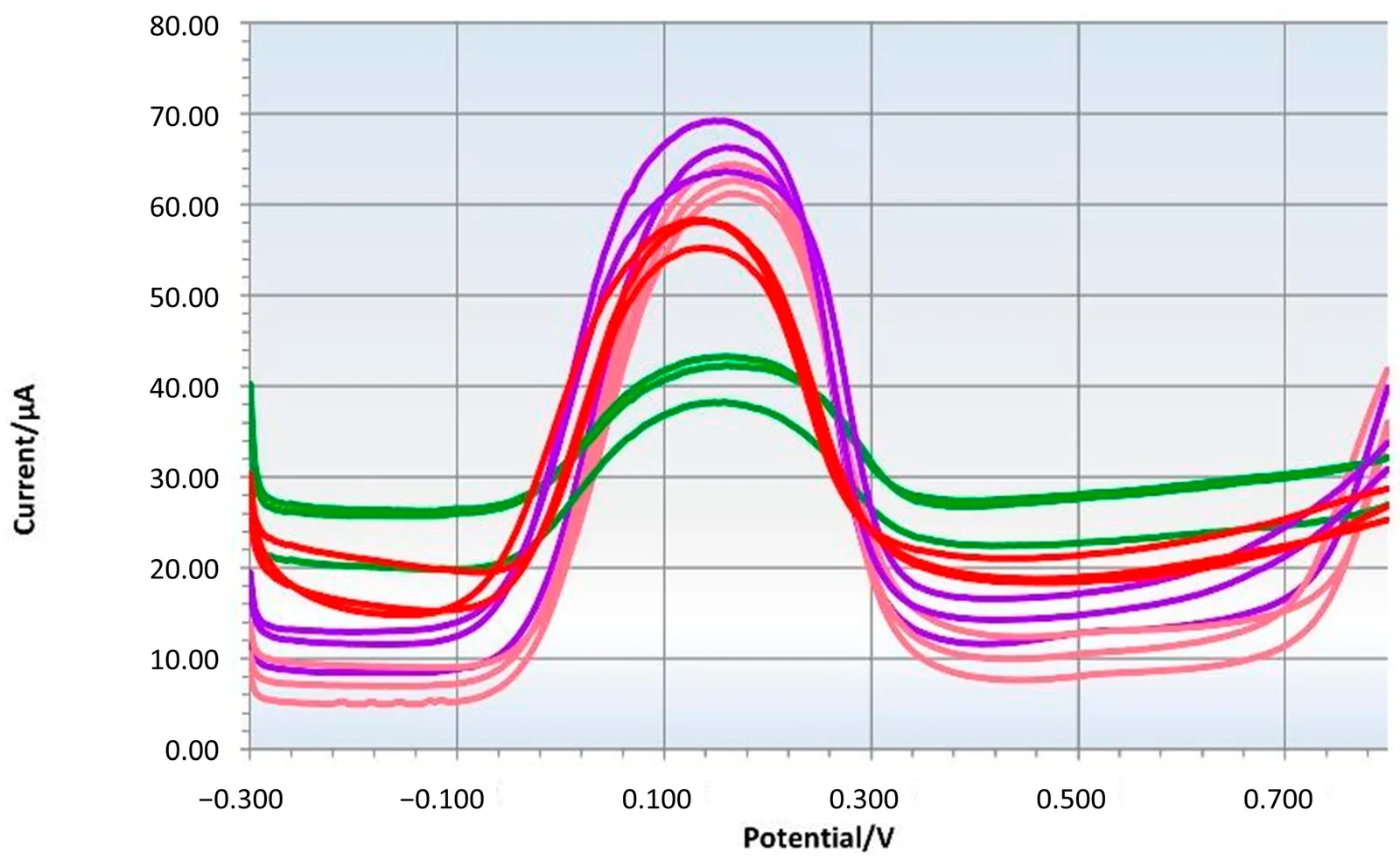
Differential pulse voltammetry (DPV) responses obtained under the evaluated experimental conditions. Conditions include: (i) SPCE-AuNS-antiSMN, immobilized anti-SMN antibody (purple); (ii) SPCE-AuNS-antiFX, immobilized anti-FX antibody (light pink); (iii) SPCE-AuNS-antiFX-SMN, SMN addition to anti-FX-modified sensors (red; negative interaction control); and (iv) SPCE-AuNS-antiSMN-SMN, SMN addition to anti-SMN-modified sensors (green; positive interaction condition).

**Figure 9:**
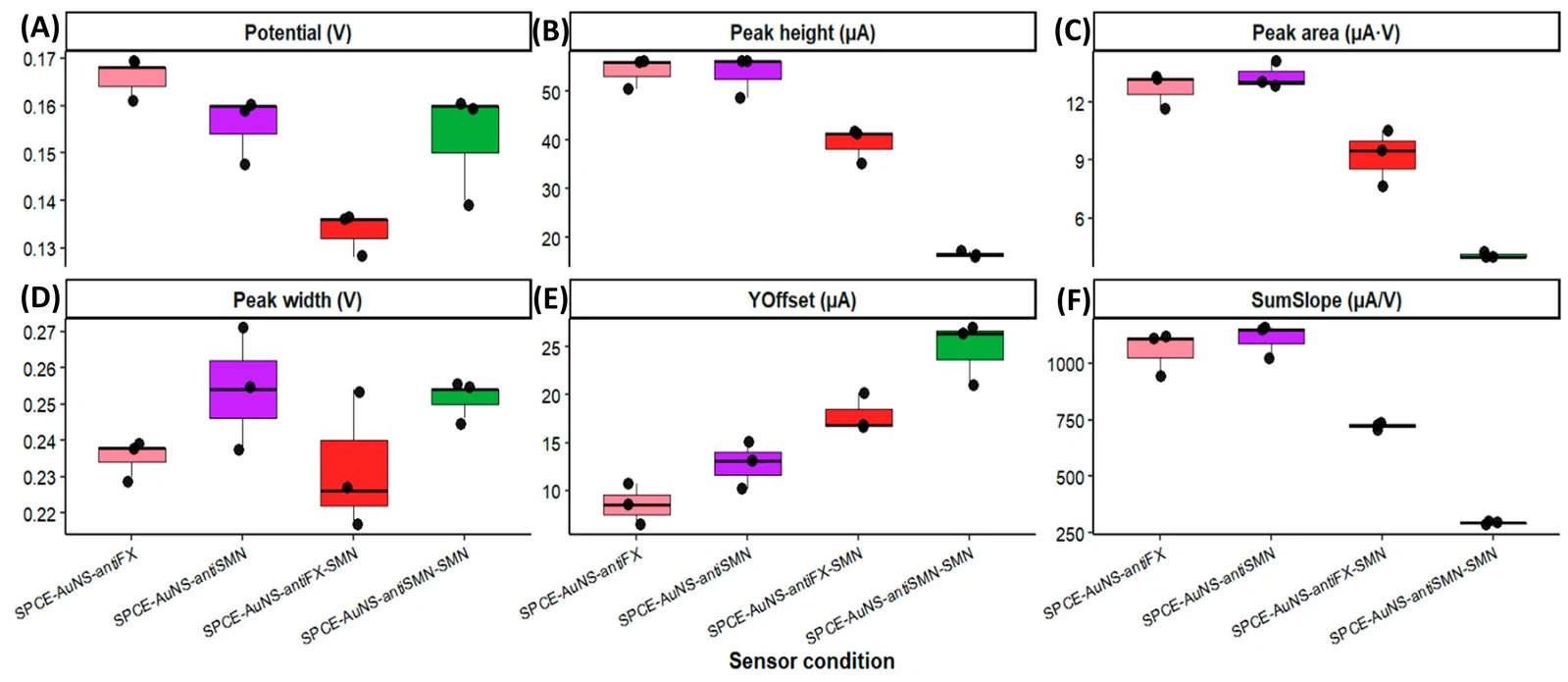
DPV electrochemical parameter distribution for SPCE-AuNS-antiSMN, SPCE-AuNS-antiFX, SPCE-AuNS-antiFX-SMN, and SPCE-AuNS-antiSMN-SMN conditions. Evaluated parameters include the potential (in (**A**)), height (in (**B**)), area (in (**C**)), width (in (**D**)), Y offset (in (**E**)) and SumSlope (in (**F**)). The SPCE-AuNS-antiSMN, SPCE-AuNS-antiFX, SPCE-AuNS-antiFX-SMN (non-specific binding, negative interaction control), and SPCE-AuNS-antiSMN-SMN (specific binding, positive interaction control), are represented in purple, light pink, red and green, respectively.

To investigate the electrochemical behavior associated with the interaction between the immobilized anti-SMN antibody and its target protein, four experimental conditions were evaluated in triplicate: (i) anti-SMN antibody immobilization (SPCE-AuNS-antiSMN), (ii) anti-FX antibody immobilization (non-specific) (SPCE-AuNS-antiFX), (iii) SMN protein addition to anti-FX-modified sensors (SPCE-AuNS-antiFX-SMN) as a non-specific condition, and (iv) SMN addition to anti-SMN-modified sensors as a specific interaction condition (SPCE-AuNS-antiSMN-SMN). This approach enabled the evaluation of the electrochemical behavior resulting from specific antigen–antibody interactions and their comparison with with non-specific interactions. Figure 8 presents the DPV responses obtained under each condition, while Figure 9 summarizes the extracted electrochemical parameters. The raw data used for these analyses are provided in Table S1 (Supplementary Information).

Regarding peak height, peak area, and Max Slope, both antibody-modified surfaces (SPCE-AuNS-antiSMN and SPCE-AuNS-antiFX) exhibited comparable electrochemical responses prior to target addition, indicating similar baseline surface properties after immobilization. However, upon the introduction of SMN protein (at a 0.36 µg/mL concentration), distinct electrochemical response patterns emerged between the specific and non-specific systems.

Before target addition, both anti-SMN- and anti-FX-modified electrodes exhibited comparable electrochemical responses, with similar peak current values (~53–55 µA), confirming consistent surface modification and baseline electron transfer properties. After SMN addition, a significant decrease in peak current was observed for both systems; however, these decreases had distinct magnitudes. The SPCE-AuNS-antiSMN system exhibited a pronounced signal suppression (~70% decrease), with peak height values ranging from 15.839 to 17.117 µA, whereas the SPCE-AuNS-antiFX system showed a more moderate reduction (~25% decrease), with height values ranging from 35.047 to 41.630 µA.

Additionally, parameters such as peak area and maximum slope followed the same trend, with the specific system showing a more substantial decrease, further supporting the hypothesis of a recognition-driven interfacial blocking mechanism. The peak area reduced from a mean of 13.3 µA·V and 12.17 µA·V for SPCE-AuNS-antiSMN and SPCE-AuNS-antiFX, respectively, to 4.0 µA·V in specific interaction (SPCE-AuNS-antiSMN-SMN) and 9.2 µA·V in non-specific interaction (SPCE-AuNS-antiFX-SMN). Also, a significant difference in Max Slope (µA/V) demonstrates a reduction from a mean of 450 µA/V after antibody immobilization to 137 µA/V in positive and 350µA/V in negative control, demonstrating a pronounced kinetic modification of the interface. The specific antigen–antibody interaction produced a pronounced decrease not only in peak current but also in peak area and SumSlope. The simultaneous reduction in these parameters indicates that the formation of the immunocomplex generated an interfacial blocking effect, reducing the accessibility of the redox probe to the electrode surface and consequently attenuating the overall electrochemical response. Specific antigen–antibody interactions may reduce the accessibility of the redox probe to the electrode surface, resulting in decreased current responses as observed by other authors [31].

From a translational perspective, the proposed sensing platform shows potential for future adaptations and application in the detection of Spinal Muscular Atrophy (SMA) screening, since this genetic disorder is characterized by reduced levels of the Survival Motor Neuron (SMN) protein. In healthy individuals, SMN protein is expressed at physiological levels sufficient to maintain motor neuron function, whereas in SMA patients, significantly decreased SMN expression is observed due to mutations or deletions in the SMN1 gene. In this context, the ability of the sensor to distinguish between specific and non-specific interactions, as well as to generate pronounced signal suppression upon SMN recognition, suggests its applicability for identifying altered SMN expression patterns.

Although the present study represents a proof-of-concept employing a fixed protein concentration (0.36 ug/mL), the observed recognition-driven electrochemical measurements and consistent response indicate that the platform may be further optimized. A limitation of the present work is the absence of concentration-dependent analyses, which prevents the determination of analytical parameters such as dynamic range, sensitivity, and limit of detection. In addition, although the electrochemical results support successful biomolecule recognition, the relative contribution of covalent immobilization and non-covalent adsorption on the AuNS-modified surface cannot be fully distinguished from the present data. Further surface-sensitive characterization techniques will be valuable to better elucidate the immobilization mechanism. Future studies involving titration curves, analytical validation, and concentration-dependent analyses, as well as evaluation in biological matrices and clinical samples will be essential to establish the analytical performance and diagnostic applicability of the proposed platform. These characteristics highlight the potential of the proposed system as a promising step for future development of point-of-care strategies aimed at early SMA detection, especially in neonatal screening programs, where rapid and decentralized detection is essential.

The main contribution of this work lies in the systematic comparison of oxygen plasma treatment and gold electrodeposition under identical experimental conditions, providing a rational basis for selecting the most suitable surface modification strategy for graphene-based SPCE immunosensors. Nevertheless, the study would benefit from extending the proof-of-concept SMN detection to oxygen-plasma-treated electrodes. This limitation should be addressed in future investigations, as such an evaluation would enable a direct comparison of the biological sensing performance of both platforms and provide additional insight into the influence of surface engineering on immunosensor performance.

## 4. Conclusions and Perspectives

This study demonstrates the benefits of applying gold electrodeposition compared to cold plasma treatment to SPCEs for immunosensor applications, demonstrating that surface engineering plays a critical role in determining the electrochemical performance of immunosensors. It demonstrates a proof-of-concept based on SMN protein detection, which could ultimately impact SMA diagnosis, screening, and monitoring. At first, the anti-SMN/SMN recognition system was validated through immunological ELISA, confirming the effectiveness of antigen–antibody binding interactions and supporting the adaptation of this system to an electrochemical sensing platform. Then, surface modification strategies were investigated to optimize sensor performance, revealing that cold plasma O_2_ treatment promotes a progressive and significant increase in hydrophilicity, while gold electrodeposition demonstrates improved electron transfer behavior, lower baseline current, and a more homogeneous electrochemical response. FESEM analysis further confirmed the successful formation and distribution of gold nanostructures (AuNSs) across the working electrode (WE) surface, supporting the enhanced electrochemical behavior observed with this modification.

The proposed SPCE-AuNS immunosensor enabled selective recognition of the antigen and differentiated between specific (anti-SMN/SMN) and non-specific (anti-FX/FX) interactions, with a pronounced blocking effect in the specific system, characterized by a reduction in peak current, peak area, and maximum slope. These findings support the applicability of the proposed platform for selective SMN recognition.

Future studies should investigate concentration-dependent responses to determine analytical sensitivity, dynamic range and limit of detection (LOD), as well as evaluate the platform performance in biological matrices and clinical samples. Complementary electrochemical techniques, including Electrochemical Impedance Spectroscopy (EIS) and Square Wave Voltammetry (SWV), may also contribute to a more comprehensive characterization of the sensing platform. Even though the present work represents a proof-of-concept, it is a promising step toward the development of a decentralized point-of-care strategy for SMA diagnosis.

## Data Availability

The original contributions presented in this study are included in the article. Further inquiries can be directed to the corresponding author.

## Supplementary Materials

The following supporting information can be downloaded at: https://www.mdpi.com/article/10.3390/s26165171/s1, Figure S1. Boxplots considering the E_pv_, E_pa_, I_pc_ and I_pa_ parameters obtained in CV to evaluate different plasma exposure times (2, 5 and 10 min are represented by orange, yellow and light brown colors); Figure S2. Boxplots considering the MinSlope and MaxSlope parameters obtained in DPV analysis to evaluate different plasma exposure times (2, 5 and 10 min are represented by orange, yellow and light brown colors); Table S1. Raw DPV data obtained from 30 SPCEs tested under 10 different experimental conditions; Table S2. Raw VC data obtained from 18 SPCEs tested under six different experimental conditions; Figure S3. Contact angle measurements of bare, AuNS-modified, and plasma-treated SPCEs. Each point represents independent measurements, while horizontal bars indicate the mean, and error bars indicate SD (n = 3) in each analysed condition. Different letters indicate statistically significant differences among groups according to one-way ANOVA followed by Tukey’s post hoc test (*p* < 0.05) and values of mean ± SD are depicted in the graph.

## Abbreviations

The following abbreviations are used in this manuscript:

SMA	Spinal Muscular Atrophy
SMN1	Survival Motor Neuron 1
RD	Rare Disease
MLPA	Multiplex Ligation Probe Amplification
POC	Point-of-Care
SPCEs	Screen-Printed Carbon Electrodes
LOD	Limit of Detection
ELISA	Enzyme-Linked Immunosorbent Assay
CV	Cyclic Voltammetry
DPV	Differential Pulse Voltammetry
HRP	Horse-Radish Peroxidase
TMB	Tetramethylbenzidine
SPCE	Screen-Printed Electrodes
CE	Counter Electrode
WE	Working Electrode
RE	Pseudo-Reference Electrode
EDC	1-ethyl-3-(3-dimethylaminopropyl)carbodiimide
NHS	N-hydroxysuccinimide
MES	2-(N-morpholino)ethanesulfonic acid
TTBS-BSA	Tris-Buffered Saline Containing Tween-20 and Bovine Serum Albumin
TBS-T	Tris-Buffered Saline with Tween-20
KCl	Potassium Chloride
HAuCl_4_	Gold(II) Chloride Hydrate
FESEM	Field Emission Scanning Electron Microscopy
AuNSs	Gold Nanostructures
EIS	Electrochemical Impedance Spectroscopy
SWV	Square Wave Voltammetry
