# Supplementary material for: Electrochemical Detection of SMN Protein by Immunosensors: The Role of Surface Modifications in Screen-Printed Carbon Electrodes
Source: Sensors (Basel). 2026 Aug 15;26(16):5171. doi: 10.3390/s26165171 (PMC13517250; doi:10.3390/s26165171)
Supplement: Supplementary file 1 [file sensors-26-05171-s001.zip › sensors-4377270-supplementary.pdf]

sensors

MDPI

Supplementary Materials

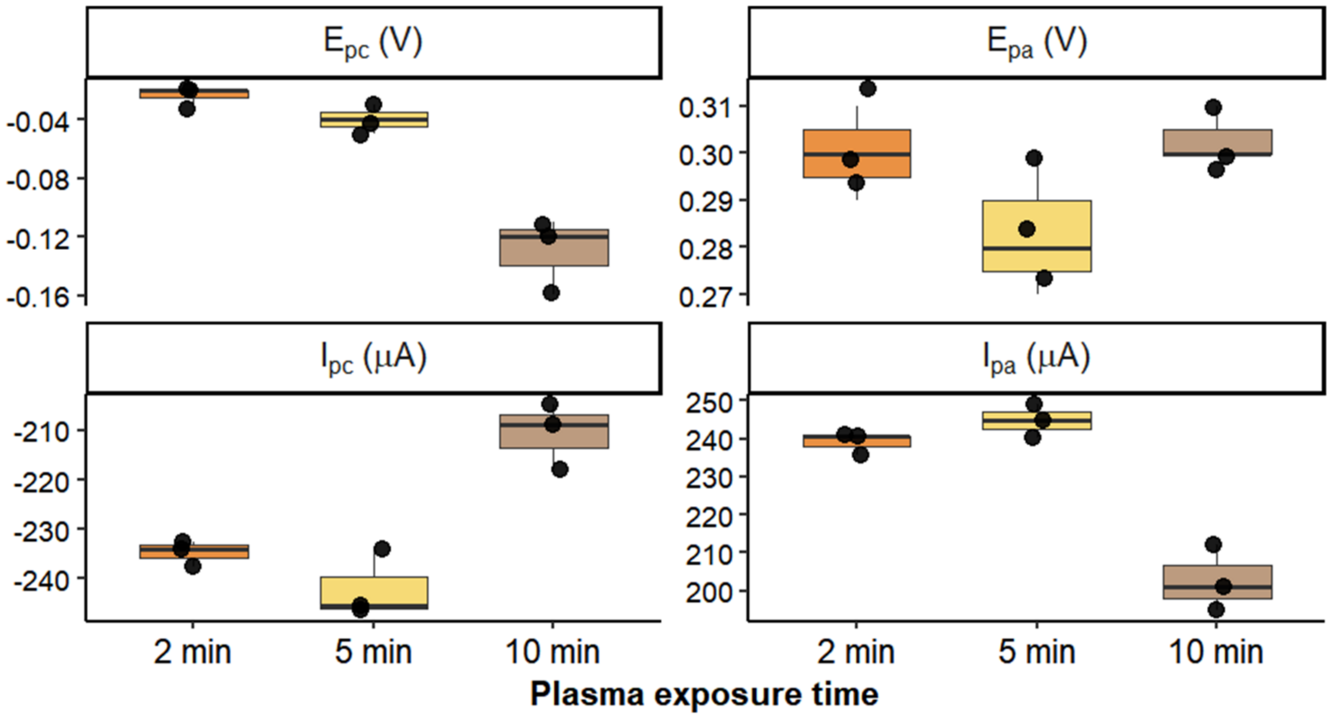

Figure S1. Boxplots considering the Epv, Epa, Ipc and Ipa parameters obtained in CV to evaluate different plasma exposure times (2, 5 and 10 min are represented by orange, yellow and light brown colors).

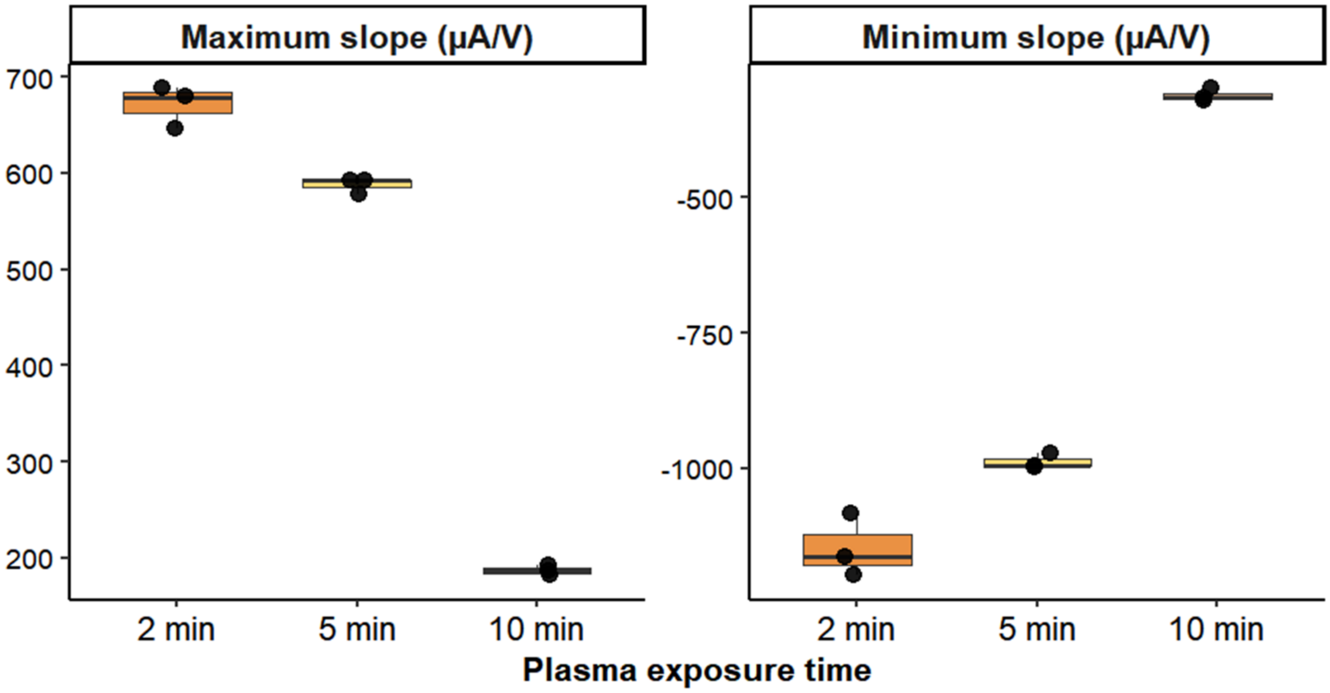

Figure S2. Boxplots considering the MinSlope and MaxSlope parameters obtained in DPV analysis to evaluate different plasma exposure times (2, 5 and 10 min are represented by orange, yellow and light brown colors).

Table S1. Raw DPV data obtained from 30 SPCEs tested under 10 different experimental conditions.

Condition	Sample	Potential (V)	Height (uA)	Area (uAV)	Width (V)	YOffset (uA)	MaxSlope (uA/V)	MinSlope (uA/V)	SumSlope (uA/V)
Basal	1	0.180	62.440	14.166	0.214	2.346	497.351	-410.985	908.336
	2	0.200	64.820	14.978	0.222	1.315	502.152	-479.394	981.547
	3	0.192	64.974	14.608	0.210	1.743	510.154	-455.730	965.883
Electrodeposition	1	0.117	99.610	19.123	0.190	2.722	854.683	-1226.723	2081.406
	2	0.128	93.171	19.255	0.206	7.812	775.047	-1072.826	1847.873
	3	0.121	97.615	18.934	0.186	9.056	823.436	-1037.616	1861.051
Plasma	1	0.117	87.720	20.095	0.234	21.359	816.479	-1196.193	2012.672
	2	0.121	69.775	16.929	0.246	26.749	574.953	-939.620	1514.573
	3	0.121	73.131	17.987	0.250	21.910	595.108	-964.580	1559.688
Plasma 2 min	1	0.140	77.428	18.451	0.242	9.159	646.319	-1083.873	1730.192
	2	0.160	80.192	19.405	0.246	8.660	678.663	-1163.241	1841.904
	3	0.156	81.590	19.515	0.242	10.209	688.244	-1197.963	1886.207
Plasma 5 min	1	0.117	70.439	17.288	0.250	18.398	593.326	-973.086	1566.411
	2	0.132	72.183	17.188	0.242	20.231	592.387	-998.002	1590.390
	3	0.117	70.118	16.928	0.246	18.324	577.953	-994.713	1572.666
Plasma 10 min	1	0.101	30.493	9.022	0.286	33.545	192.375	-320.642	513.017

	2	0.113	28.095	8.374	0.286	34.402	182.308	-297.634	479.942
	3	0.117	28.808	8.670	0.290	31.863	186.079	-315.087	501.167
Cab SMN	1	0.160	56.179	13.053	0.238	10.142	469.950	-677.693	1147.643
	2	0.160	48.628	12.845	0.270	15.008	407.750	-614.616	1022.366
	3	0.148	56.138	14.142	0.254	13.092	469.523	-685.346	1154.869
Cab FX	1	0.168	50.493	11.651	0.230	10.678	420.297	-521.795	942.092
	2	0.160	55.866	13.195	0.238	8.510	461.815	-655.171	1116.986
	3	0.168	56.222	13.310	0.238	6.398	468.156	-641.175	1109.331
Positive Complex	1	0.160	15.839	3.949	0.254	26.421	128.694	-166.965	295.658
	2	0.160	16.298	3.941	0.246	26.963	136.671	-162.862	299.533
	3	0.140	17.117	4.244	0.254	21.028	145.852	-139.780	285.632
Negative Complex	1	0.128	41.630	10.533	0.254	16.642	332.175	-374.154	706.329
	2	0.136	35.047	7.604	0.218	20.164	360.238	-374.367	734.606
	3	0.136	41.282	9.484	0.226	16.828	359.649	-365.179	724.828

Table S2. Raw VC data obtained from 18 SPCEs tested under six different experimental conditions.

Condition	Sample	E_pc (Cathodic peak potential)	I_pc (Cathodic peak current)	E_{pa} (Anodic peak potential)	I_{pa} (Anodic peak current)	ΔE
Basal	1	-0.080	-60.493	0.400	129.801	0.480
	2	-0.060	-65.118	0.390	130.740	0.450
	3	-0.070	-79.206	0.360	138.044	0.430
Electrodeposition	1	-0.020	-201.482	0.280	231.209	0.300
	2	-0.020	-204.775	0.290	239.211	0.310
	3	-0.030	-214.352	0.270	241.012	0.300
Plasma	1	-0.060	-253.252	0.270	249.027	0.330
	2	-0.060	-238.908	0.280	238.053	0.340
	3	-0.040	-245.140	0.270	246.332	0.310
Plasma 2 min	1	-0.020	-232.475	0.300	235.663	0.320
	2	-0.020	-234.116	0.310	240.504	0.330
	3	-0.030	-237.745	0.290	240.960	0.320
Plasma 5 min	1	-0.030	-234.000	0.300	240.177	0.330
	2	-0.050	-245.584	0.270	244.792	0.320
	3	-0.040	-246.558	0.280	249.055	0.320
Plasma 10 min	1	-0.160	-204.661	0.300	194.751	0.460
	2	-0.110	-217.938	0.300	212.051	0.410
	3	-0.120	-208.895	0.310	201.095	0.430

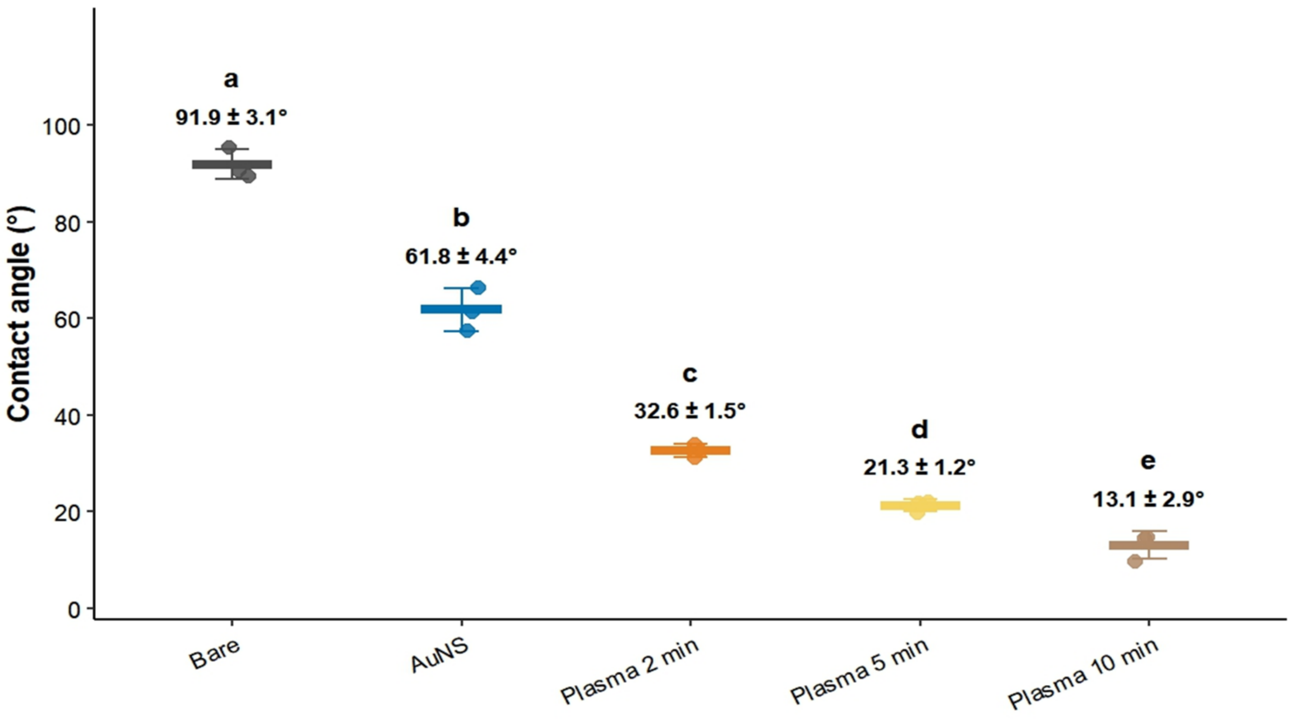

Figure S3. Contact angle measurements of bare, AuNS-modified, and plasma-treated SPCEs. Each point represents independent measurements,while horizontal bars indicate the mean, and error bars indicate SD (n = 3) in each analysed condition. Different letters indicate statistically significant differences among groups according to one-way ANOVA followed by Tukey's post hoc test ($p < 0.05$) and values of mean ± SD are depicted in the graph.
